# Supplementary material for: Nanopore sequencing of intact aminoacylated tRNAs
Source: Nat Commun. 2025 Aug 20;16:7781. doi: 10.1038/s41467-025-62545-9 (PMC12368100; doi:10.1038/s41467-025-62545-9)
Supplement: Supplementary file 1 — Supplementary Information [file 41467_2025_62545_MOESM1_ESM.pdf]

SUPPLEMENTARY INFORMATION FOR WHITE RADAKOVIC ET AL

SUPPLEMENTARY TABLES

Supplementary Table 1. Yeast strains

Strain	Genotype	Source	Publication
S288C	*MATa/α ho/ho ura-352/ura-d*		
BY4741	*MATa his3Δ1 leu2Δ0 ura3Δ0 met15Δ0*	Open Biosystems	
JMW 009	BY4741 *trm8Δ::NatR trm4Δ::KanR*	E. Phizicky	PMID 18443146
YJD236-1	BY4741 *trm8Δ::NatR trm4Δ::KanR met22Δ::HygR*	E. Phizicky	PMID 18443146
EG328-1A	*MATα ura3-52 leu2 trp1*	R. Wek	PMID 19546227
WY798	*MATα URA3 LEU2 TRP1*	R. Wek	PMID 19546227
WY795	*MATα URA3 leu2 TRP1*	R. Wek	PMID 19546227

Supplementary Table 2. Oligonucleotide sequences

Oligonucleotide Name	Use in this study	Sequence
Acceptor stem mimic (fluorescein labeled)	tRNA mini-substrate experiments	/56-FAM/rUrUrCrCrCrGrCrUrCrCrA
Acceptor stem mimic (complement)	tRNA mini-substrate experiments	/5Phos/rGrCrGrGrGrArA
Hairpin adapter	tRNA mini-substrate experiments	/5phos/rGrCrUrCrGrArArArGrArGrCrUrGrGrA

tRNA (Gly-GCC)	Flexizyme-charged synthetic tRNA experiments	/5Phos/rGrCrGrGrGrArArUrArGrCrUrCrArGrUrUrGrGrUrArGrArGrCrArCrGrArCrCrUrUrGrCrCrArArGrGrUrCrGrGrGrGrUrCrGrCrGrArGrUrUrCrGrArGrUrCrUrCrGrUrUrUrCrCrCrGrCrUrCrCrA
Splint (also the 5' adapter)	Flexizyme-charged synthetic tRNA experiments	rCrCrUrArArGrArGrCrArArGrArArGrArArGrCrCrUrGrGrA
Revised splint (also the 5' adapter)	Flexizyme-charged synthetic tRNA experiments	CCTAAGAGCAAGAAGAAGCrCrUrGrGrA
Activated 3' adapter	Flexizyme-charged synthetic tRNA experiments	/5phos/rGrGrCrUrUrCrUrUrCrUrUrGrCrUrCrUrUrArGrGrArArArArArArArArArAAAA
dFx Flexizyme	Flexizyme-charged synthetic tRNA experiments	rGrGrArUrCrGrArArArGrArUrUrUrCrCrGrCrArUrCrCrCrCrGrArArArGrGrGrUrArCrArUrGrGrCrGrUrUrArGrGrU
5' splint adapter (universal)	Biological tRNA experiments	CCTAAGAGCAAGAAGAAGCrCrUrGrGrN
Charged 3' adapter (chemical ligation to aa-tRNA)	Biological tRNA experiments	/5Phos/rGrGrCrUrUrCrUrUrCrUrUrGrCrUrCrUrUrCrCrArArCrCrUrUrGrCrCrUrUAAAAAAAAAA
Uncharged 3' adapter (enzymatic ligation to tRNA)	Biological tRNA experiments	/5Phos/rGrGrCrUrUrCrUrUrCrUrUrGrCrUrCrUrUrArUrGrGrArArGrGrUrArGrGrCAAAAAAAAAA
Gly-GCC-3'-exon probe	Yeast northern probe	TGCGCAAGCCCGGAATCGAA
His-GUG-5'-exon probe	Yeast northern probe	GTACTAACCACTATACTAAG
Pro-UUG-5' exon probe	Yeast northern probe	CCCAAAGCGAGAATCATACCACTAGACCACACGCCC
Leu-CAA-3'-exon probe	Yeast northern probe	AGAGATTCGAACTCTTGCAT
Asn-GUU-3'-exon probe	Yeast northern probe	ACCCCAGTGAGGGTTGAACTCA
Cys-CGA-3'-exon probe	Yeast northern probe	AGCTCGCACTCAGGATCGAACTA

Phe-GAA-5'-exon probe	Yeast northern probe	CTTCAGTCTGGCGCTCTCCCAACTGAGCTAAATCCGC
Tyr-GUA-3'-exon probe	Yeast northern probe	TCTCCCGGGGGCGAGTCGAA
Val-AAC-3'-exon probe	Yeast northern probe	TGGTGATTTCGCCCAGGA
Trp-CCA-5'-exon probe	Yeast northern probe	CGATTTGGAGTCGAAAGCTCTACCATTGAGCCACCGC
Glu-UUC-3'-exon probe	Yeast northern probe	CTCCGATACGGGGAGTCGAAC
Ser-GCU-3'-exon probe	Yeast northern probe	AGGATTCGAACCTGCGCAGGT
Ile-UAU-3'-exon probe	Yeast northern probe	TGCTCGAGGTGGGGTTTGAA
Gln-UUG-3'-exon probe	Yeast northern probe	AGGTCCTACCCGGATTCGAAC
Thr-UGU-3'-exon probe	Yeast northern probe	TGCCACCTGTCAGAAT
Arg-UCU-3'-exon probe	Yeast northern probe	CACTCACGATGGGGGTC
Asp-GUC-3'-exon probe	Yeast northern probe	CGACGGGGAATTGAACCCCGAT
Lys-UUU-3'-exon probe	Yeast northern probe	ACCCCTGACATTTCGG
iMet-CAU-3'-exon probe	Yeast northern probe	GCGCCGCTCGGTTTCGATCC
Ala-UGC-3'-exon probe	Yeast northern probe	TGGACGCAACCGGAATCGAA
5S-yeast probe	Yeast northern probe	CTCGGTCAGGCTCTTACCAG

SUPPLEMENTARY FIGURES

Figure S1

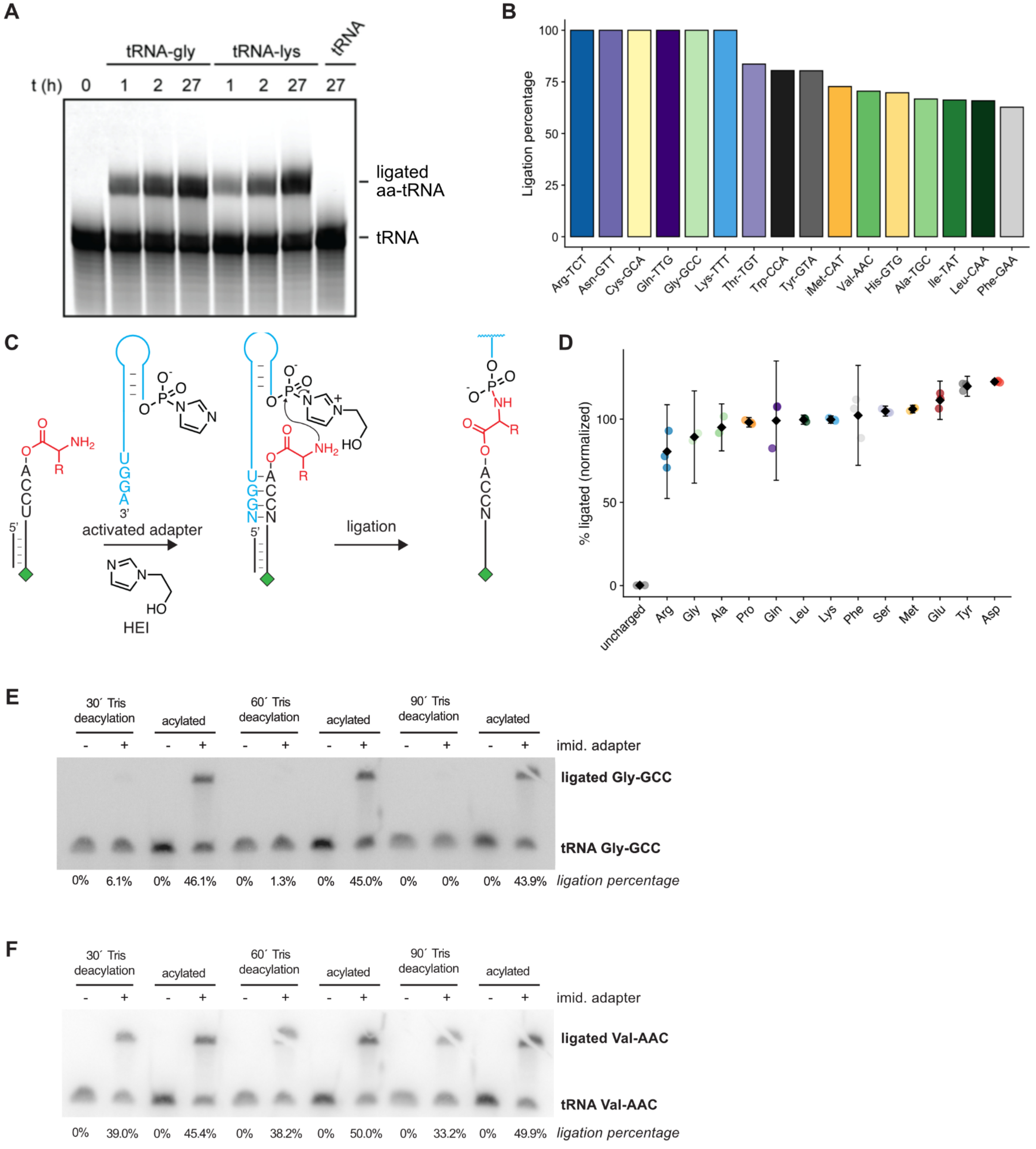

Supplementary Figure 1.

(**A**) Chemical ligation of Flexizyme-charged synthetic tRNA in the absence of catalyst for 0, 1, 2, or 27 hours. The analytical 16 % denaturing gel was stained with SYBR gold to visualize the RNA. The upper band represents adapter-ligated, aminoacylated tRNA, while the unligated lower bands may include both charged and uncharged synthetic tRNA molecules depending on ligation efficiency (see Supplementary Fig. 2).

(**B**) Densitometry-based quantifications of the percent of aminoacylated tRNA shifted upon chemical ligation for the budding yeast tRNAs visualized on the acidic northern in **Fig. 1B**, after stripping and reprobing for the indicated isodecoders. Data represents a single value per tRNA species.

(**C**) Schematic of a 16 nt tRNA minisubstrate bearing a fluorophore (FAM, green diamond) undergoing chemical ligation to 5′-phosphorimidazolide activated hairpin adapter in the presence of the catalyst 1-(2-hydroxyethyl)imidazole (HEI).

(**D**) Normalized levels of ligation product for fluorescently labeled tRNA minisubstrate in (**B**) aminoacylated with the indicated amino acids using Flexizyme and reacted with the activated hairpin adapter in the presence of HEI for 90 minutes.

(**E**) Chemical-charging northern displaying a time course of budding yeast tRNA deacylated in Tris pH 9 for 30-90 minutes, with an untreated ("acylated") control loaded next to each timepoint. Each sample was split in half prior to reaction in the presence (+) or absence (-) of the activated 3′ adapter to chemically ligate aminoacylated tRNA. The membrane was probed for tRNA-Gly-GCC.

(**F**) The same northern membrane as in (**E**), stripped and reprobed for tRNA-Val-AAC.

Fig. S2 (A-D)

A

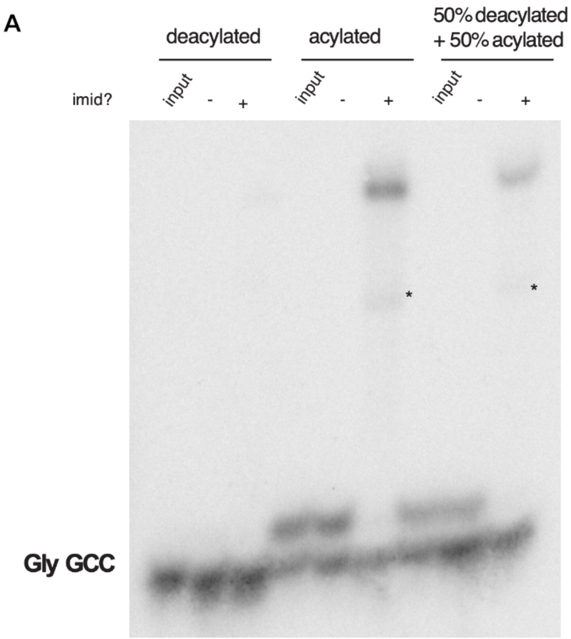

Chemiligated			0.1%			34.4%			11.3%
*						4%			1.4%
Acylated				47.5%	42.4%		25.4%	17.6%	
Deacylated	100%	100%	99.9%	52.5%	57.6%	61.6%	74.6%	82.4%	87.3%

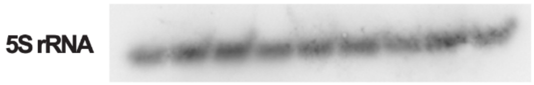

1.00	1.44	1.52	1.28	1.36	1.33	1.19	1.41	1.09

B

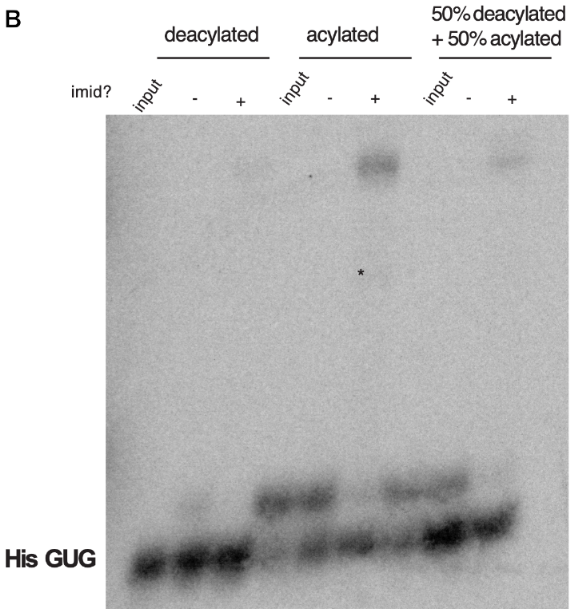

Chemiligated			4%			23%			9.9%
*						2%			
Acylated		9%		43%	38%	11%	31%	22%	0.1%
Deacylated	100%	91%	96%	57%	62%	64%	69%	78%	89%

C

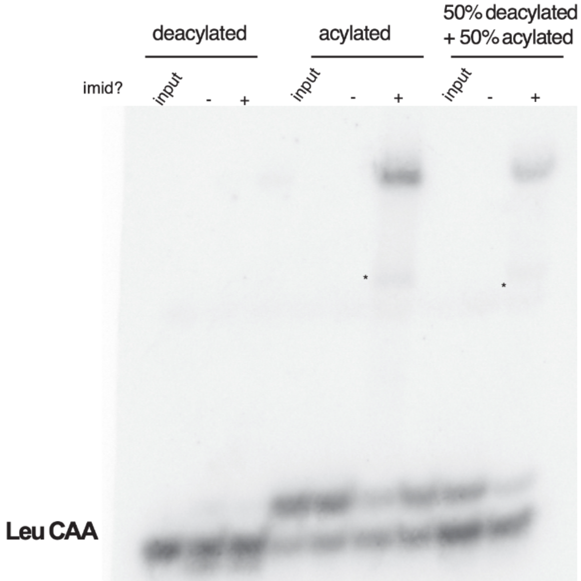

Chemiligated			2%			34%			19%
*						6%			3%
Acylated		15%	10%	55%	53%	21%	44%	34%	14%
Deacylated	100%	91%	88%	45%	47%	39%	56%	66%	64%

D

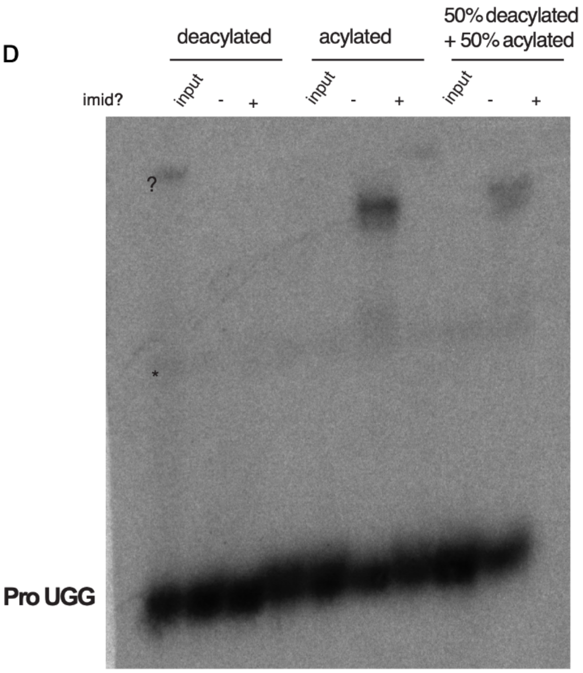

?	5%					2%			
Chemiligated					25%				15%
*	2.1%		0.3%	1.3%	1%	0.5%	0.7%	0.8%	3%
Unligated	93%	99.7%	100%	98.7%	70%	97%	99%	99.2%	82%

Fig. S2 (E-H)

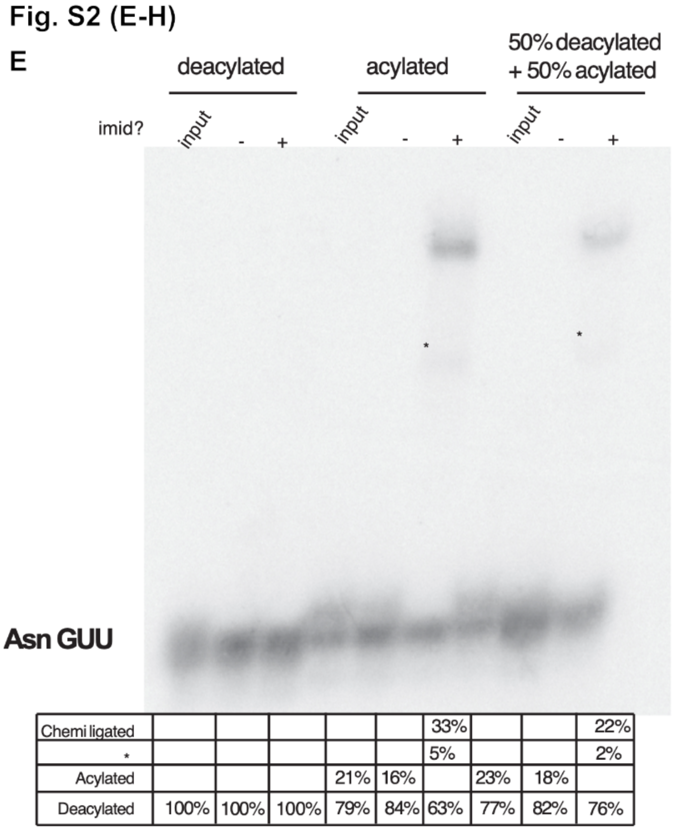

Chemi ligated						33%			22%
*						5%			2%
Acylated				21%	16%		23%	18%	
Deacylated	100%	100%	100%	79%	84%	63%	77%	82%	76%

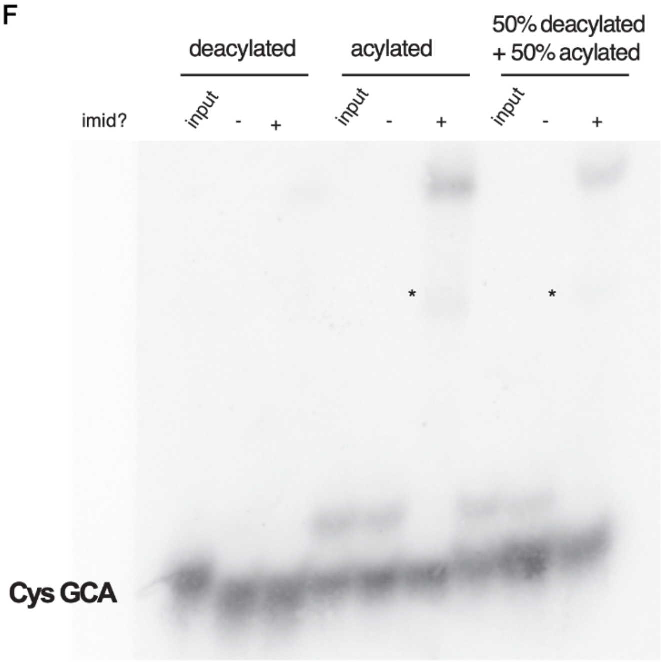

Chemi ligated			0.6%			21%			12.3%
*						3%			0.7%
Acylated				17%	14%		13%	10%	
Deacylated	100%	100%	99.4%	83%	86%	75%	87%	90%	87%

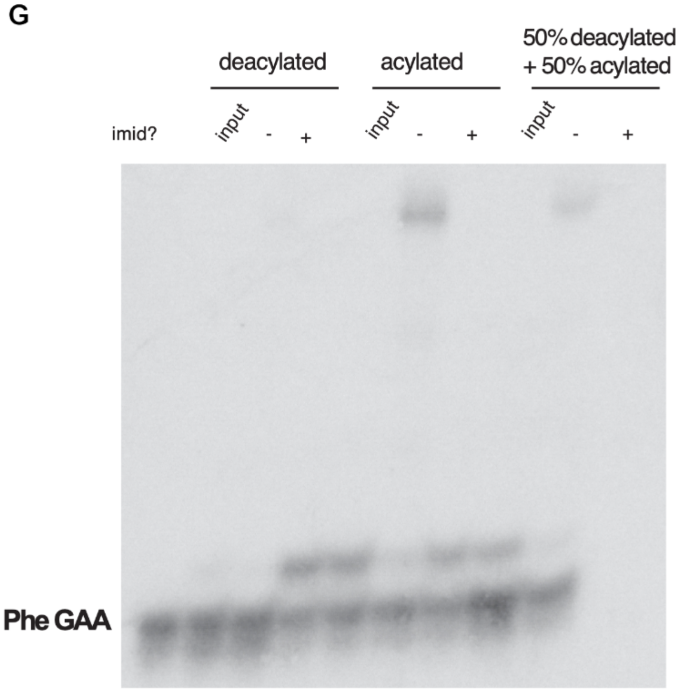

Chemi ligated			2%			20%			10%
Acylated		5%	5%	32%	31%	12%	26%	20%	8%
Deacylated 1	58%	51%	49%	40%	41%	40%	43%	48%	54%
Deacylated 2	42%	44%	44%	28%	28%	28%	31%	32%	28%

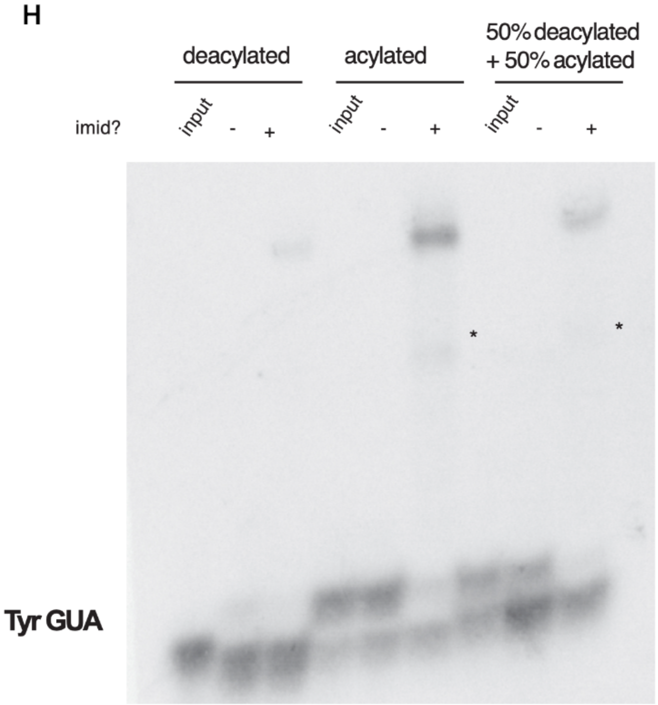

Chemi ligated			6%			38%			19%
*						6%			2%
Acylated		4%	4%	46%	46%	11%	35%	26%	5%
Deacylated	100%	96%	90%	54%	54%	45%	65%	74%	74%

Fig. S2 (I-L)

I

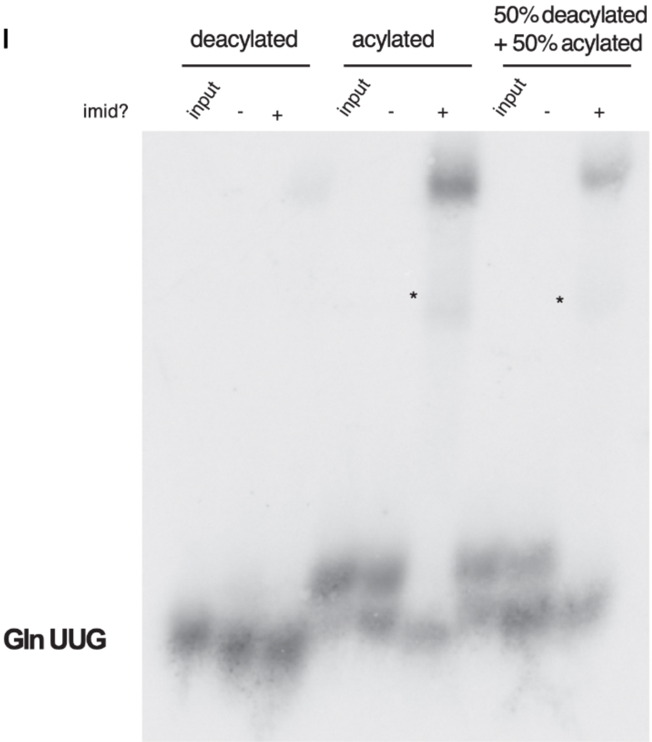

Chemiligated			4%			48%			33%
*						8%			4%
Acylated		7%		51%	47%		46%	36%	
Deacylated	100%	93%	96%	49%	53%	44%	54%	64%	63%

J

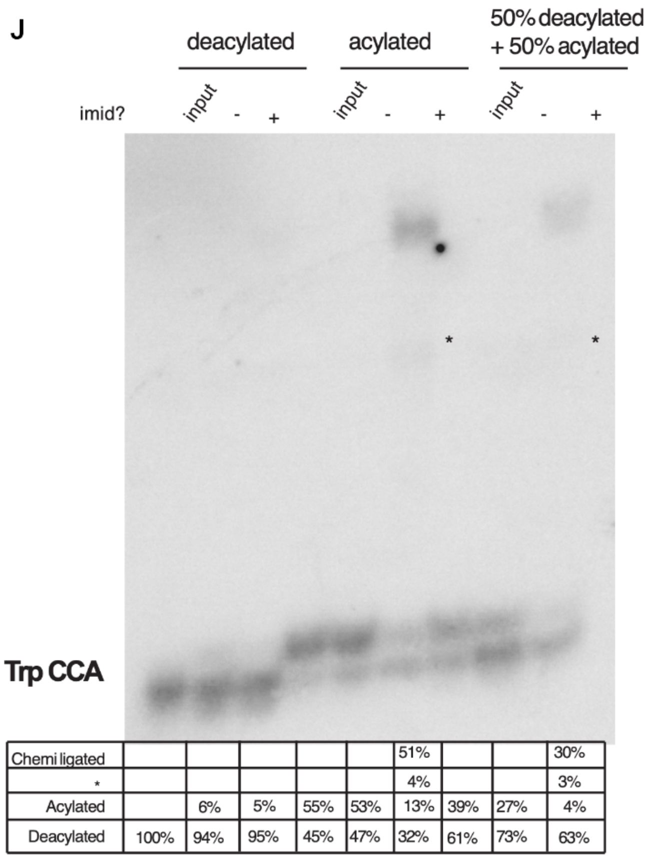

Chemiligated						51%			30%
*						4%			3%
Acylated		6%	5%	55%	53%	13%	39%	27%	4%
Deacylated	100%	94%	95%	45%	47%	32%	61%	73%	63%

K

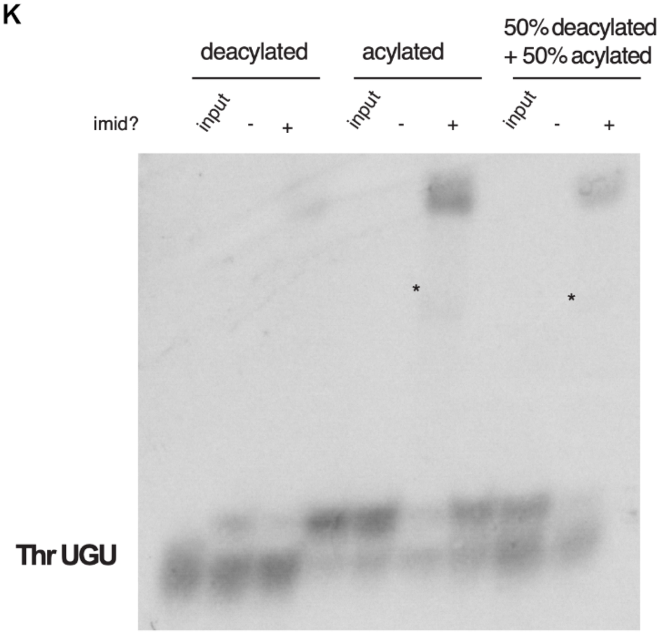

Chemiligated			10%			49%			30%
*						6%			3%
Acylated		17%	11%	53%	52%	11%	41%	33%	9%
Deacylated	100%	83%	79%	47%	48%	34%	59%	67%	58%

L

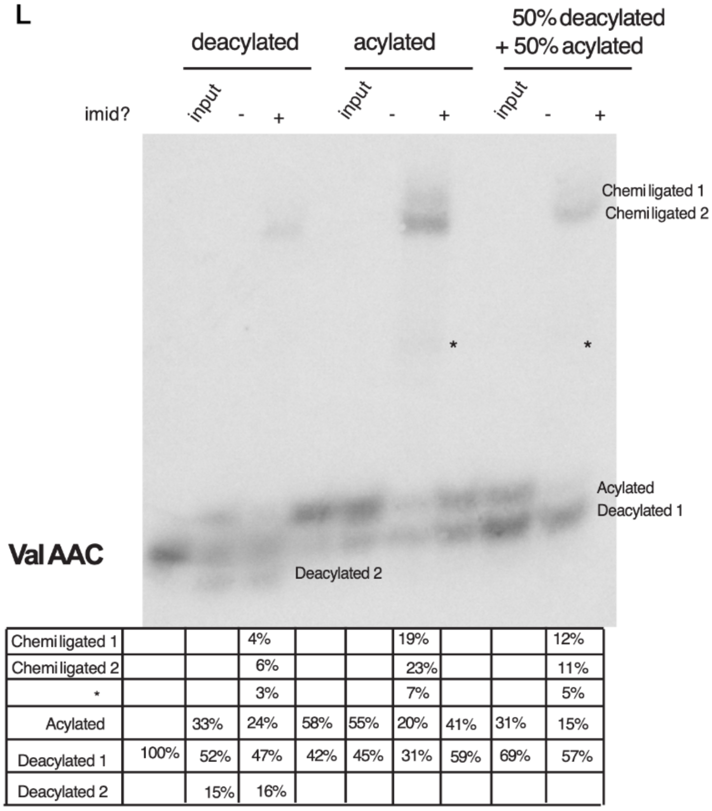

Chemiligated 1			4%			19%			12%
Chemiligated 2			6%			23%			11%
*			3%			7%			5%
Acylated		33%	24%	58%	55%	20%	41%	31%	15%
Deacylated 1	100%	52%	47%	42%	45%	31%	59%	69%	57%
Deacylated 2		15%	16%						

Fig. S2 (M-P)

M

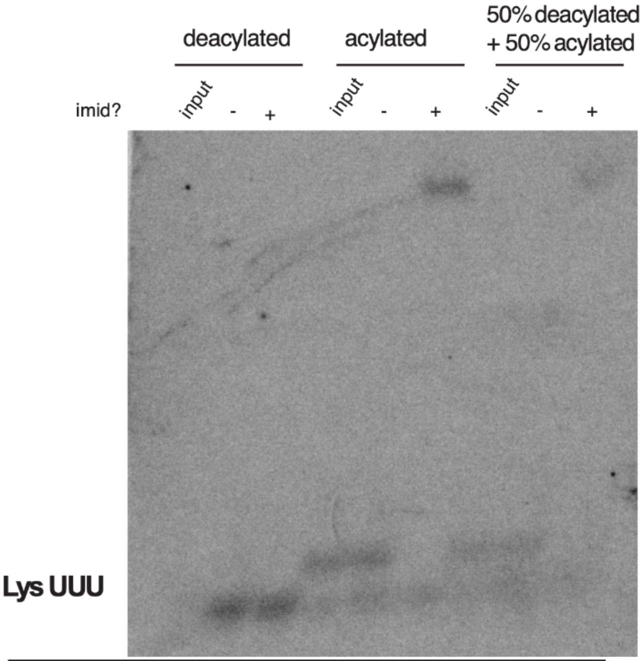

Chemi ligated						57%			37%
Acylated				65%	62%		63%	53%	
Deacylated	100%	100%	100%	35%	38%	43%	37%	47%	63%

N

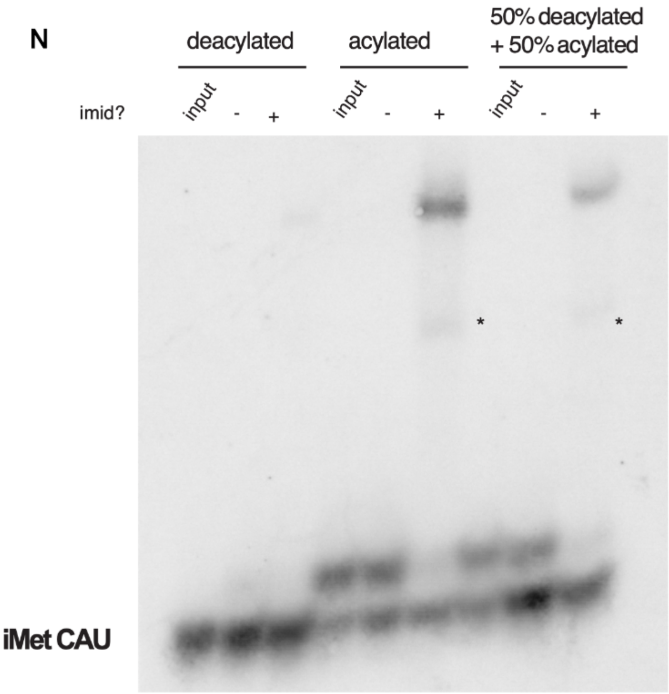

Chemi ligated			2%			33%			19%
*						4%			2%
Acylated		9%	11%	44%	43%	14%	36%	26%	4%
Deacylated	100%	91%	87%	56%	57%	49%	64%	74%	75%

O

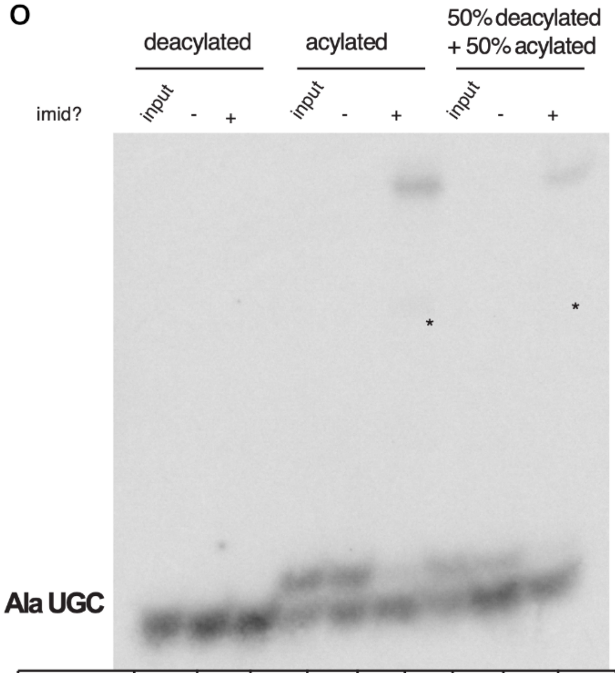

Chemi ligated						19%			11%
*						2%			1%
Acylated				37%	34%	11%	23%	14%	3%
Deacylated	100%	100%	100%	63%	66%	68%	77%	86%	85%

P

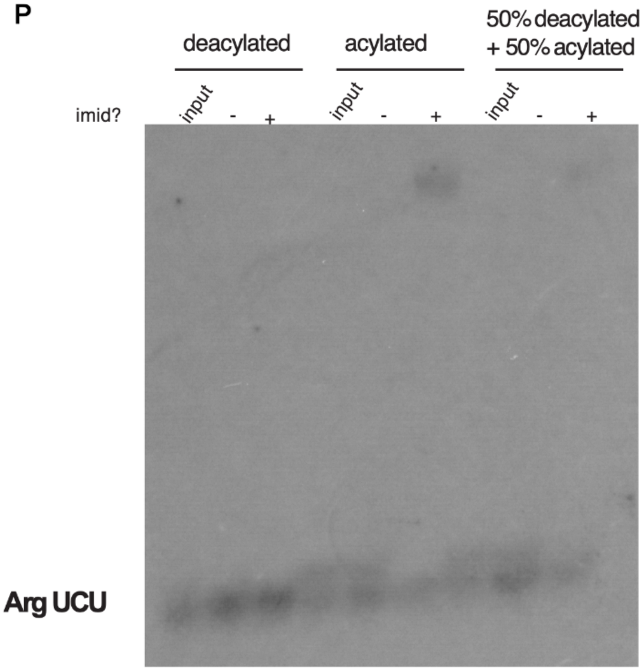

Chemi ligated						74%			53%
Acylated				55%	52%		60%	47%	
Deacylated	100%	100%	100%	45%	48%	26%	40%	53%	47%

Fig. S2 (Q-T)

Q

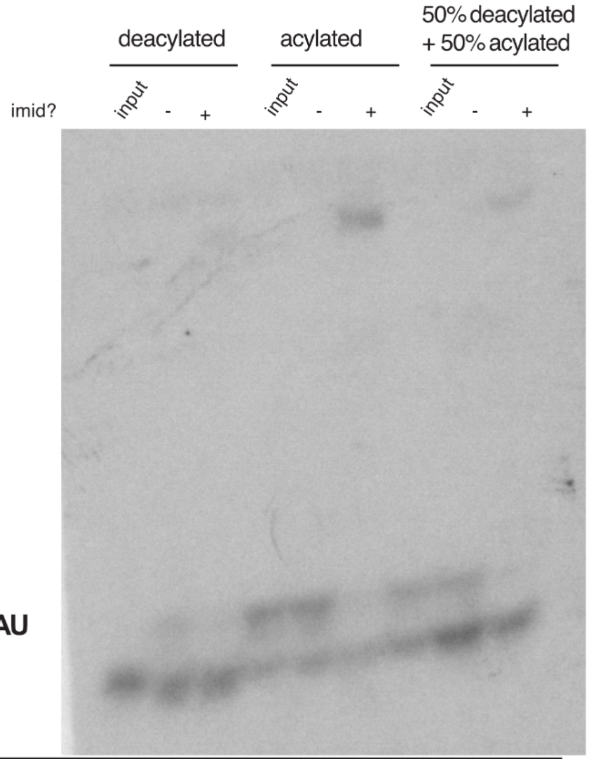

Ile UAU

Chemi ligated						36%			12%
Acylated		17%	14%	39%	44%	19%	38%	31%	20%
Deacylated	100%	83%	86%	61%	56%	45%	62%	69%	68%

R

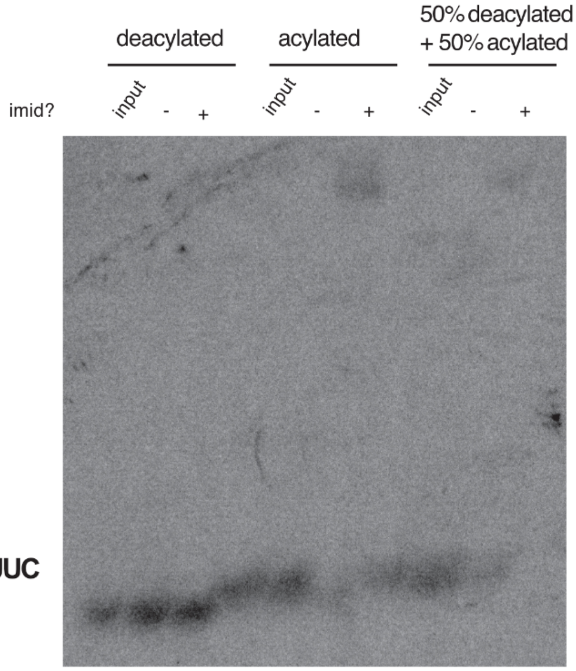

Glu UUC

Chemi Ligated						35%			0%
Unligated	100%	100%	100%	100%	100%	65%	100%	100%	100%

S

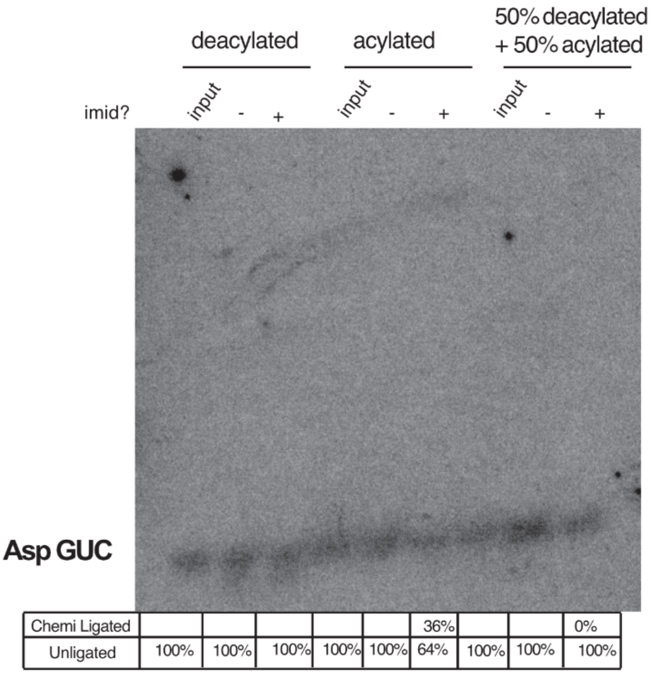

Asp GUC

Chemi Ligated						36%			0%
Unligated	100%	100%	100%	100%	100%	64%	100%	100%	100%

T

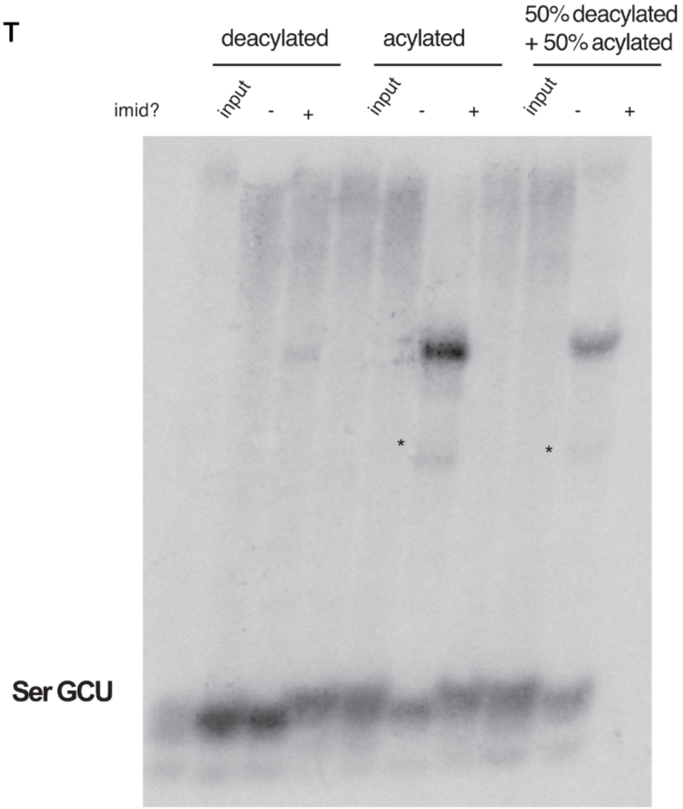

Ser GCU

Chemi Ligated			12%			57%			42%
*						6%			5%
Unligated	100%	100%	88%	100%	100%	37%	100%	100%	53%

Supplementary Figure 2.

Densitometric quantification of acylated and deacylated tRNA species from wild-type (BY4741) *S. cerevisiae* in the presence (+) or absence (-) of activated 3′ adapter, compared to tRNA input only (I). Lanes 1-3 represent a chemically deacylated control, lanes 4-6 untreated yeast tRNA, and lanes 7-9 a 50/50 mixture of the previous inputs. Asterisks indicate presumed ligation intermediates, while question marks indicate putative background ligation to incompletely deacylated tRNA species. (**A**) Densitometric quantification of the full membrane shown in **Fig. 1B**, probed for tRNA-Gly-GCC. This membrane was then stripped and re-probed with oligonucleotides complementary to budding yeast (**B**) His-GUG, (**C**) Leu-CAA, (**D**) Pro-UGG, (**E**) Asn-GUU, (**F**) Cys-GCA, (**G**) Phe-GAA, (**H**) Tyr-GUA, (**I**) Gln-UUG, (**J**) Trp-CCA, (**K**) Thr-UGU, (**L**) Val-AAC, (**M**) Lys-UUU, (**N**) iMet-CAU, (**O**) Ala-UGC, (**P**) Arg-UCU, (**Q**) Ile-UAU, (**R**) Glu-UUC, (**S**) Arg-GUC, and (**T**) Ser-GCU isodecoders. The intensities of each band relative are indicated as a relative percentage per lane in the tables below each panel.

Figure S3

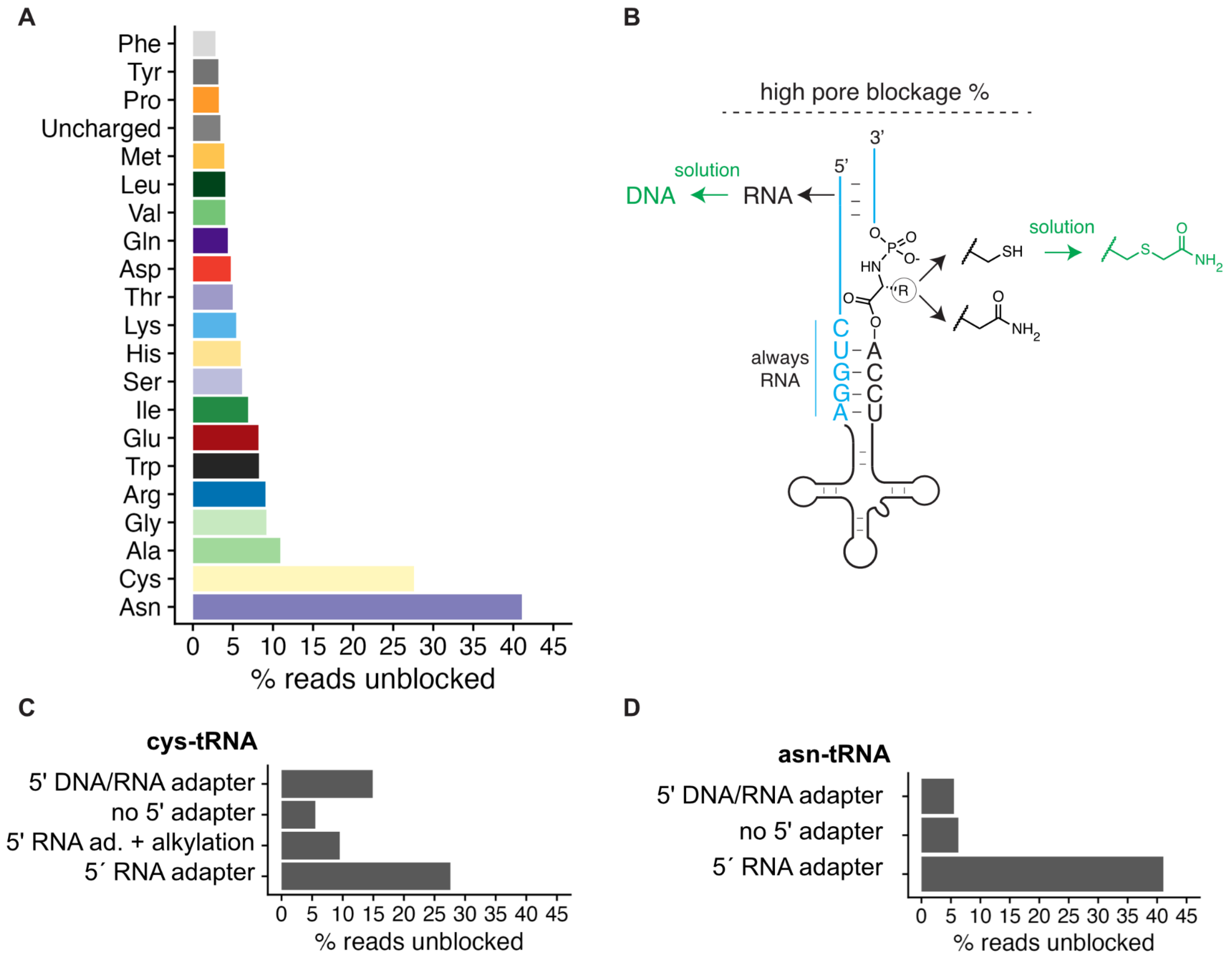

Supplementary Figure 3.

(**A**) Percentage of reads with "unblock" end status in nanopore sequencing libraries from synthetic tRNA chemically ligated to an imidazolated 3′ RNA adapter and enzymatically ligated to a 5′ RNA adapter.

(**B**) Schematic depicting experimental strategies tested for their effects on Asn- and Cys-tRNA pore blocking rates, including incorporation of a DNA/RNA hybrid 5′ adapter, alkylation of cys-tRNA, or hydrolysis of Flexizyme-charged asn-tRNA.

(**C**) Percent of Cys-tRNA reads with "unblock" status using each of the relevant strategies above, as well as omission of 5′ adapter.

(**D**) Equivalent percentage of "unblocked" Asn-tRNA reads for all three approaches tested.

Figure S4

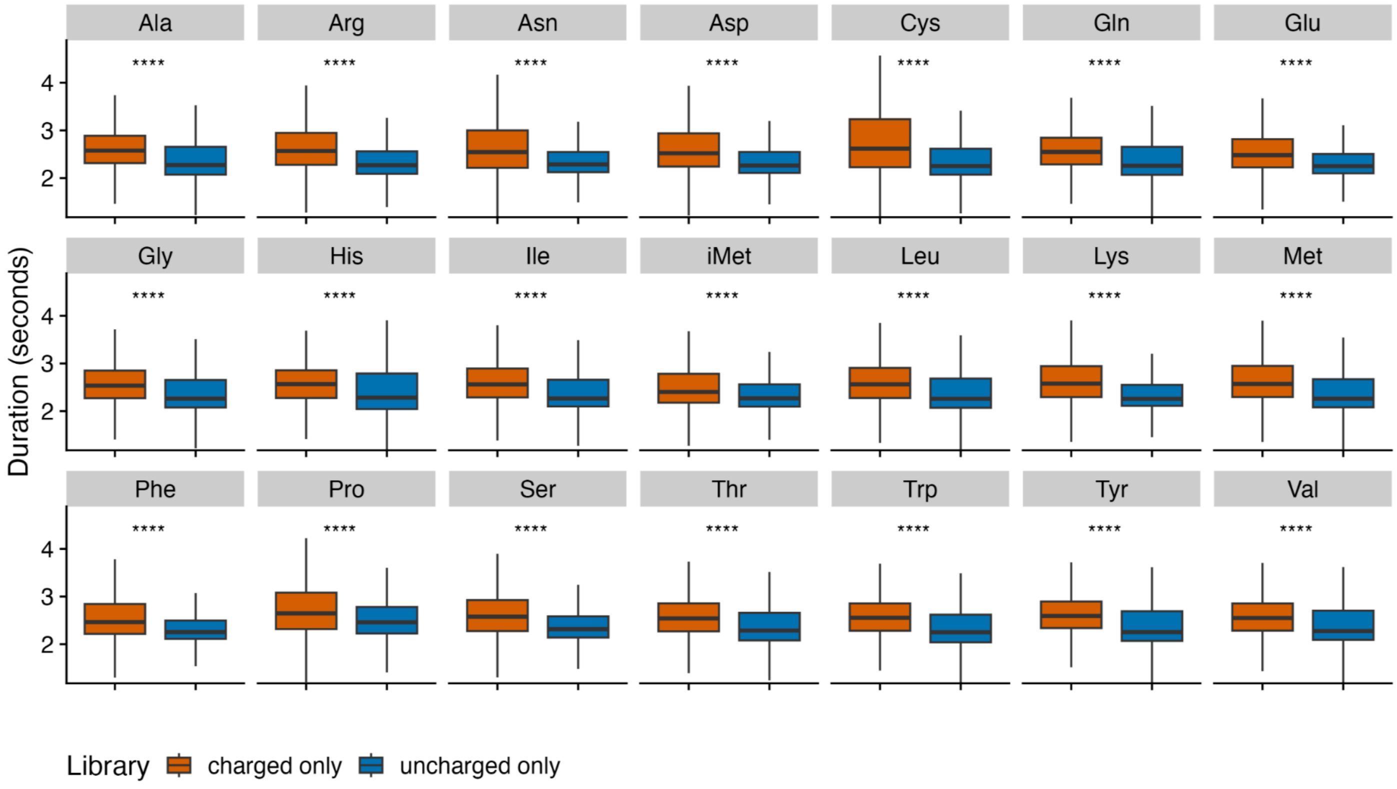

Supplementary Figure 4.

tRNA translocation time by isodecoder in budding yeast tRNA sequencing libraries prepared using only chemical ligation to imidazole-charged adaptors ("charged only", orange), or libraries where tRNA was first chemically deacylated followed by enzymatic ligation with T4 RNL2 ("uncharged only", blue). The y-axis displays the translocation duration for the entire read in seconds. Statistical significance between the distributions in each library was assessed using the Wilcoxon test, with significance levels indicated by asterisks above each comparison (****$p < 0.0001$).

Figure S5

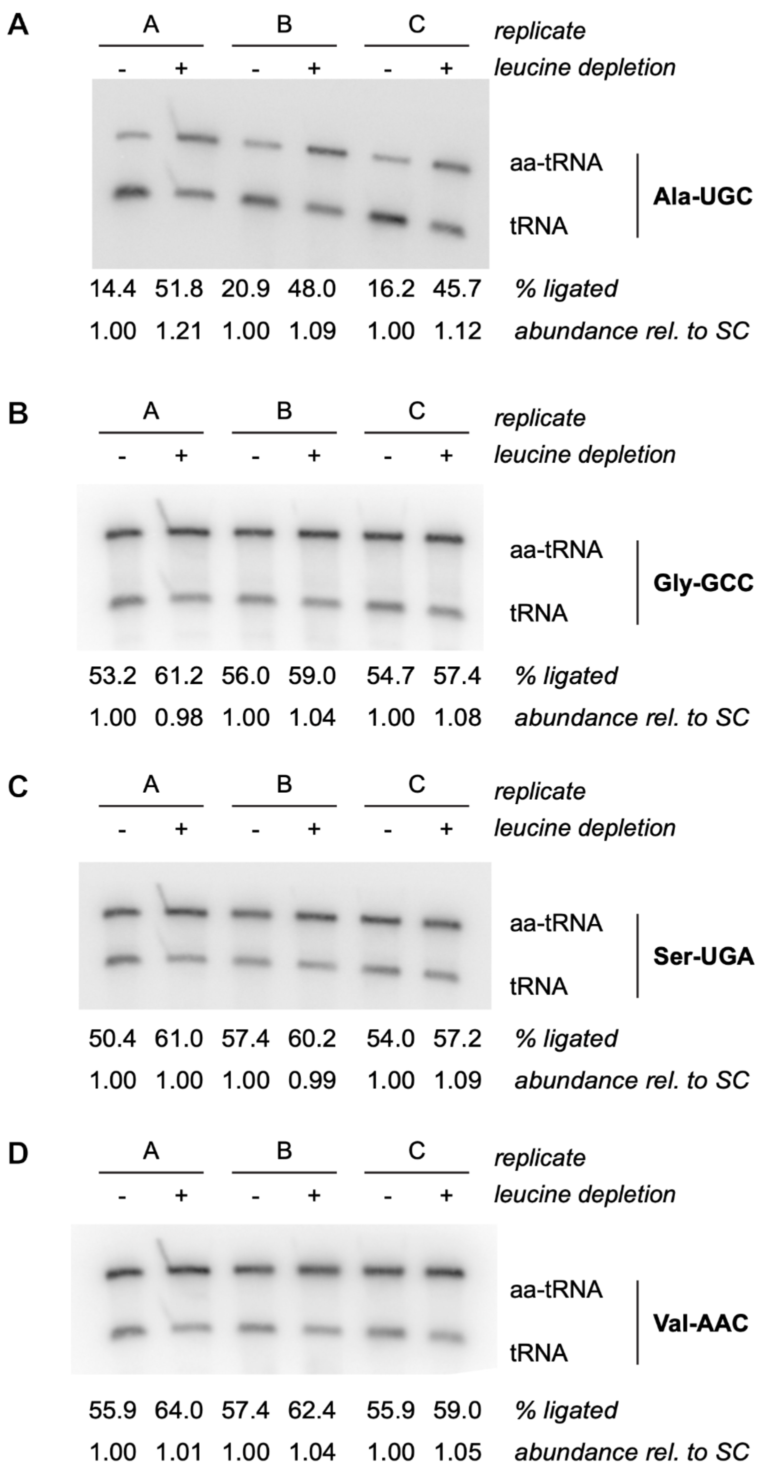

Supplementary Figure 5.

Chemical-charging northern analysis of chemically ligated tRNA to visualize relative aminoacylation levels for (**A**) Ala-UGC, (**B**) Gly-GCC, (**C**) Ser-UGA, and (**D**) Val-AAC isodecoders in a budding yeast leucine auxotroph upon 15 minutes of leucine depletion, as measured by the percent of tRNA chemically ligated in each lane (upper

band), with the percent-ligated tRNA per sample quantified under each lane. Signals were normalized to 5S rRNA probe abundance shown in **Fig. 3B**; relative abundances represent within-replicate normalized levels of total tRNA (with the sample grown in complete media normalized to 1.0 and compared to the abundance for each leucine-starved sample). Panels display the results from three independent biological replicates.

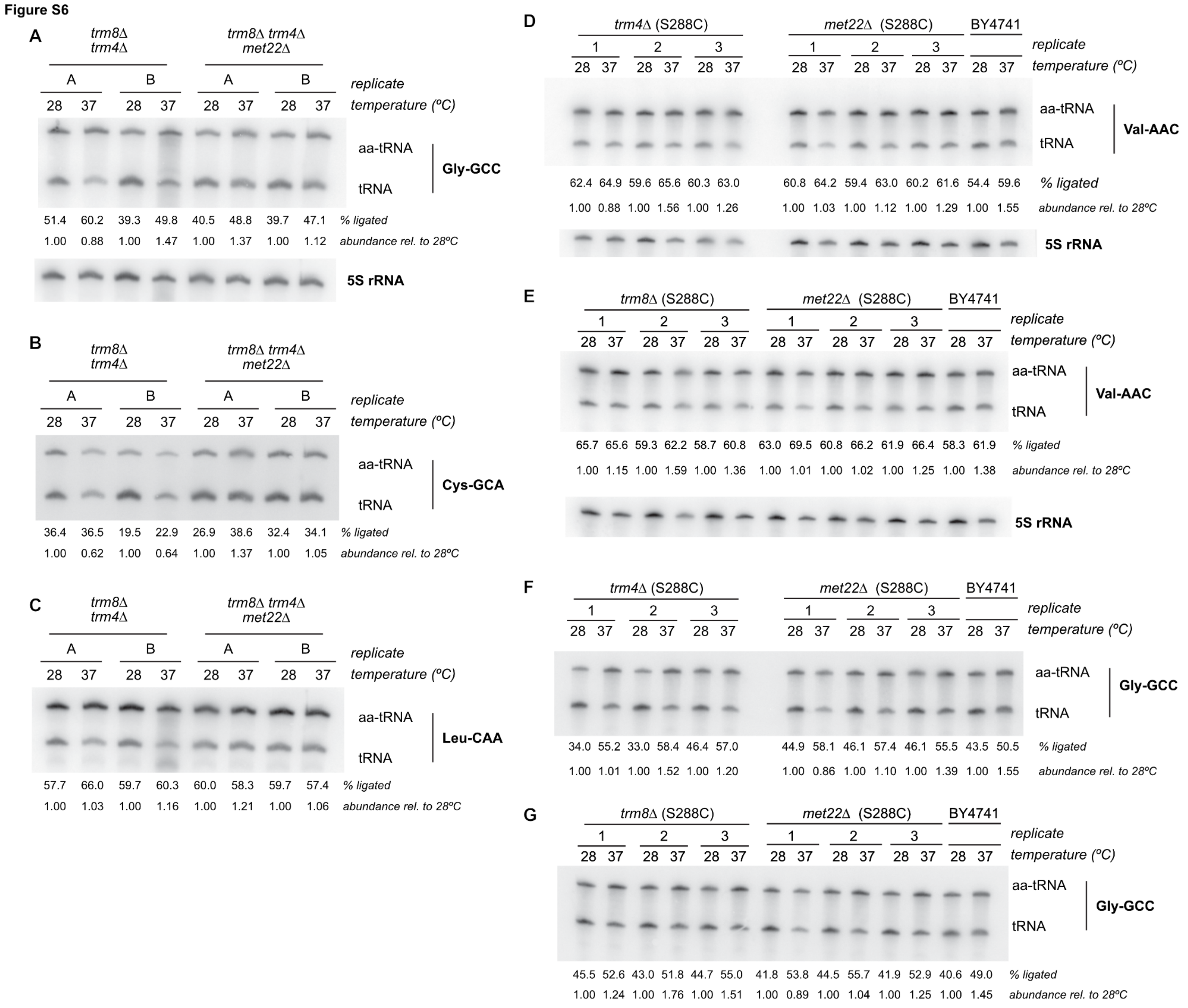

Supplementary Figure 6.

Chemical-charging northern analysis of chemically ligated tRNA from budding yeast with single, double, or triple genetic deletions affecting rapid tRNA decay (RTD), compared to a wild-type control (BY4741). Independent biological replicates (two for panels A-C; three for panels D-G) were grown at permissive (28ºC) and nonpermissive (37ºC) temperatures for three hours. The percent of chemically ligated tRNA (upper band) is indicated below each lane, with a 5S rRNA probe as a loading control. The relative abundances represent within-replicate normalized levels of total tRNA (with the samples grown at 28ºC normalized to 1.0 and compared to the abundance for a matched sample shifted to 37ºC). (**A**) Co-deletion of *TRM8* and *TRM4* and triple deletion with *MET22* as in **Fig. 3E**, but with membrane re-probed for Gly-GCC, (**B**) Cys-GCA, or (**C**) Leu-CAA. The inset below panel (**A**) contains the same membrane re-probed for 5S ribosomal RNA as a loading control. (**D**) Single deletions of *TRM4* and *MET22*, with membrane probed for Val-AAC. The inset below shows the same membrane re-probed for 5S rRNA as a loading control. (**E**) Single deletions of *TRM8* and *MET22*, with membrane probed

for Val-AAC. The inset below shows the same membrane re-probed for 5S rRNA. (**F**) Single deletions of *TRM4* and *MET22*, where the membrane from (**D**) has been re-probed for Gly-GCC. (**G**) Single deletions of *TRM8* and *MET22*, where the membrane from (**E**) has been re-probed for Gly-GCC.

Figure S7

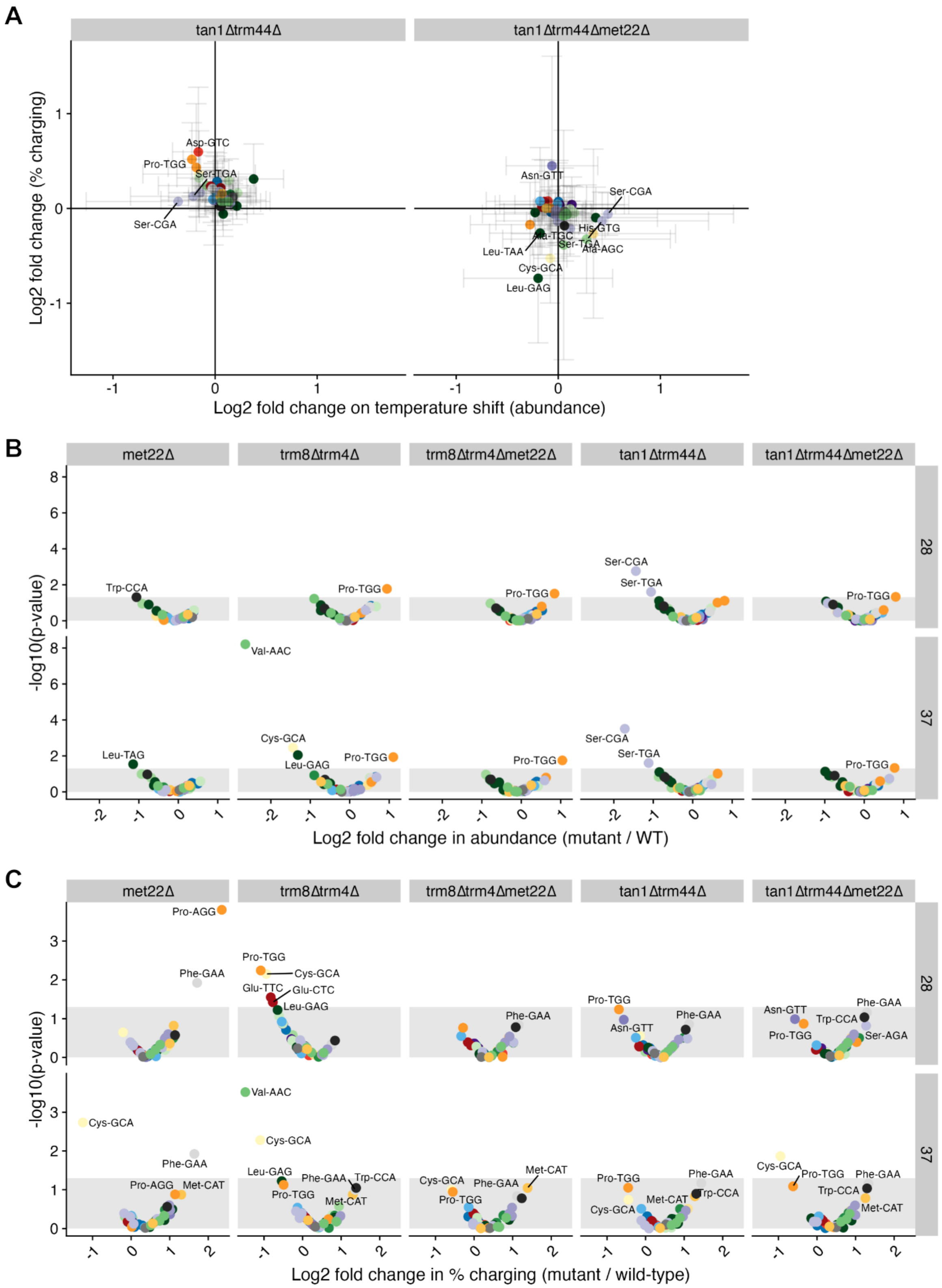
A
tan1Δtrm44Δ
tan1Δtrm44Δmet22Δ
Log2 fold change (% charging)
Log2 fold change on temperature shift (abundance)
Asp-GTC
Pro-TGG
Ser-TGA
Ser-CGA
Asn-GTT
His-GTG
Ala-TGC
Leu-TAA
Ala-AGC
Cys-GCA
Leu-GAG
B
met22Δ
trm8Δtrm4Δ
trm8Δtrm4Δmet22Δ
tan1Δtrm44Δ
tan1Δtrm44Δmet22Δ
28
37
-log10(p-value)
Log2 fold change in abundance (mutant / WT)
Trp-CCA
Pro-TGG
Leu-TAG
Val-AAC
Cys-GCA
Leu-GAG
C
Log2 fold change in % charging (mutant / wild-type)
Pro-AGG
Phe-GAA
Glu-TTC
Glu-CTC
Asn-GTT
Ser-AGA
Met-CAT

Supplementary Figure 7. Additional analysis of aa-tRNA-seq data from RTD strains grown at permissive (28ºC) and nonpermissive (37ºC) temperatures.

(A) Log_2 fold change in tRNA abundance and tRNA charging percent (charged reads) in the RTD-sensitive budding yeast strain *tan1Δ trm44Δ* and the RTD-resistant strain *tan1Δ trm44Δ met22Δ* after 3 hours growth at the nonpermissive or permissive temperatures. Points represent the mean values for each tRNA isodecoder across 3 biological replicates, with error bars spanning the standard deviation.

(B) Volcano plot comparing the mean fold change in tRNA abundance for all RTD-sensitive and resistant strains sequenced in this study, with two-sided Z-test p-values on the y-axis and the grey box indicating the α threshold. Each panel contains data from triplicate sequencing of two RTD sensitive budding yeast strains (*trm8Δ trm4Δ* and *tan1Δ trm44Δ*) as well as the corresponding RTD-resistant strains (*trm8Δ trm4Δ met22Δ* and *tan1Δ trm44Δ met22Δ*), and a control strain with a *MET22* disruption.

(C) Volcano plot comparing the mean fold change in tRNA aminoacylation for all RTD-sensitive and resistant strains sequenced in this study. Each panel contains data from triplicate sequencing of two RTD sensitive budding yeast strains (*trm8Δ trm4Δ* and *tan1Δ trm44Δ*) as well as the corresponding RTD-resistant strains (*trm8Δ trm4Δ met22Δ* and *tan1Δ trm44Δ met22Δ*), and a control strain with a *MET22* disruption. The y axis contains two-sided Z-test p-values, and the grey box indicates the α threshold.

Figure S8

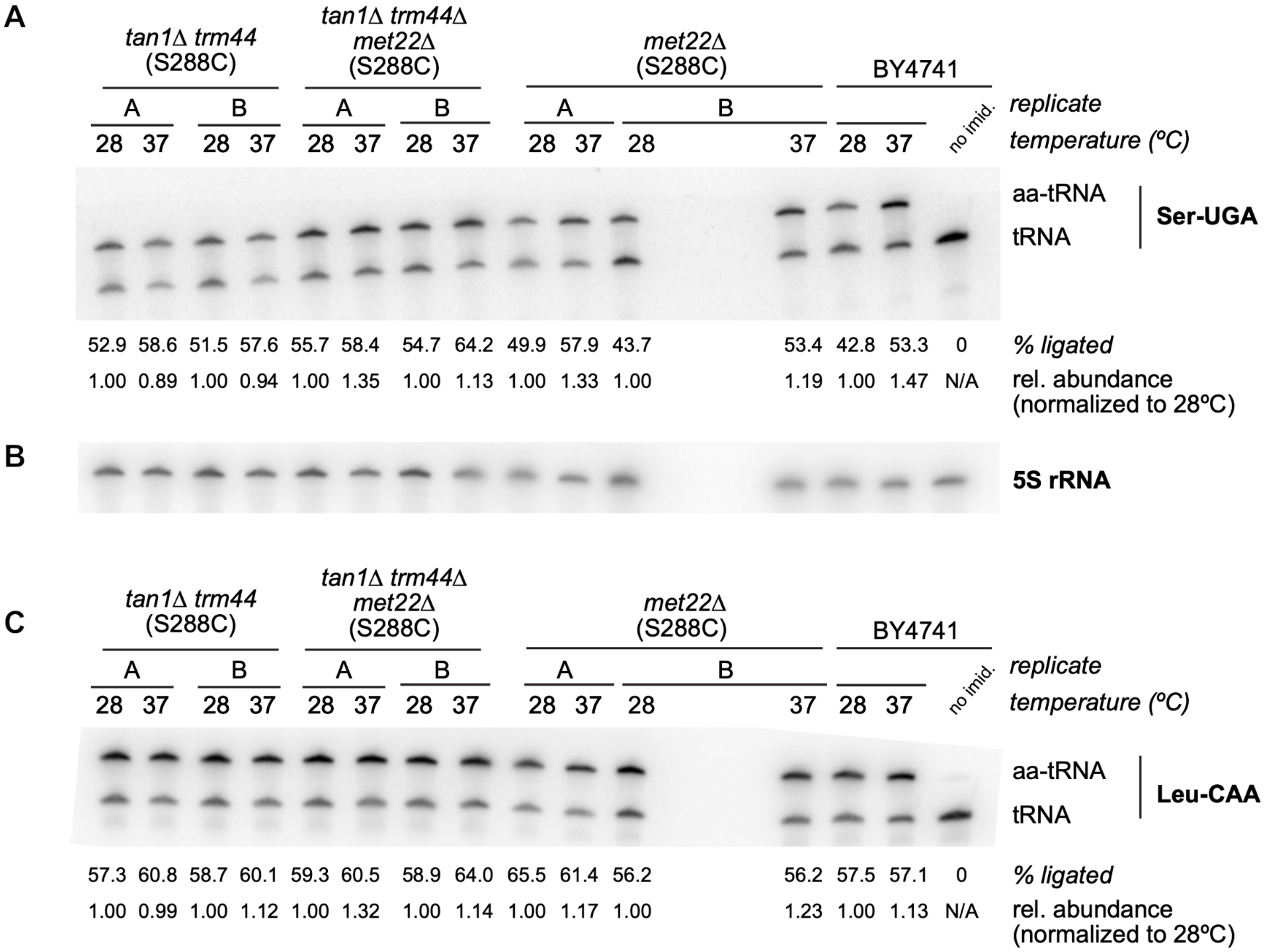

Supplementary Figure 8.

Chemical-charging northern analysis of chemically ligated tRNA from two independent biological replicates of the RTD-sensitive budding yeast strain *tan1Δ trm44Δ* and the RTD-resistant strain *tan1Δ trm44Δ met22Δ* grown at permissive (28ºC) and nonpermissive (37ºC) temperatures for three hours. tRNA extracted from BY4741 yeast were loaded as an additional control in the last 3 lanes, with the final lane containing tRNA from the 28ºC sample where the imidazolated 3′-adapter was not added to the chemical ligation reaction. (**A**) The percent of chemically ligated Ser-UGA tRNA (upper band) is indicated below each lane. The relative abundances represent within-replicate normalized levels of total tRNA (with the samples grown at 28ºC normalized to 1.0 and compared to the abundance for a matched sample shifted to 37ºC). (**B**) Reprobing of the same membrane using a 5S rRNA probe as a loading control. (**C**) Additional reprobing of the same membrane with an oligonucleotide complementary to Leu-CAA tRNA.

Figure S9

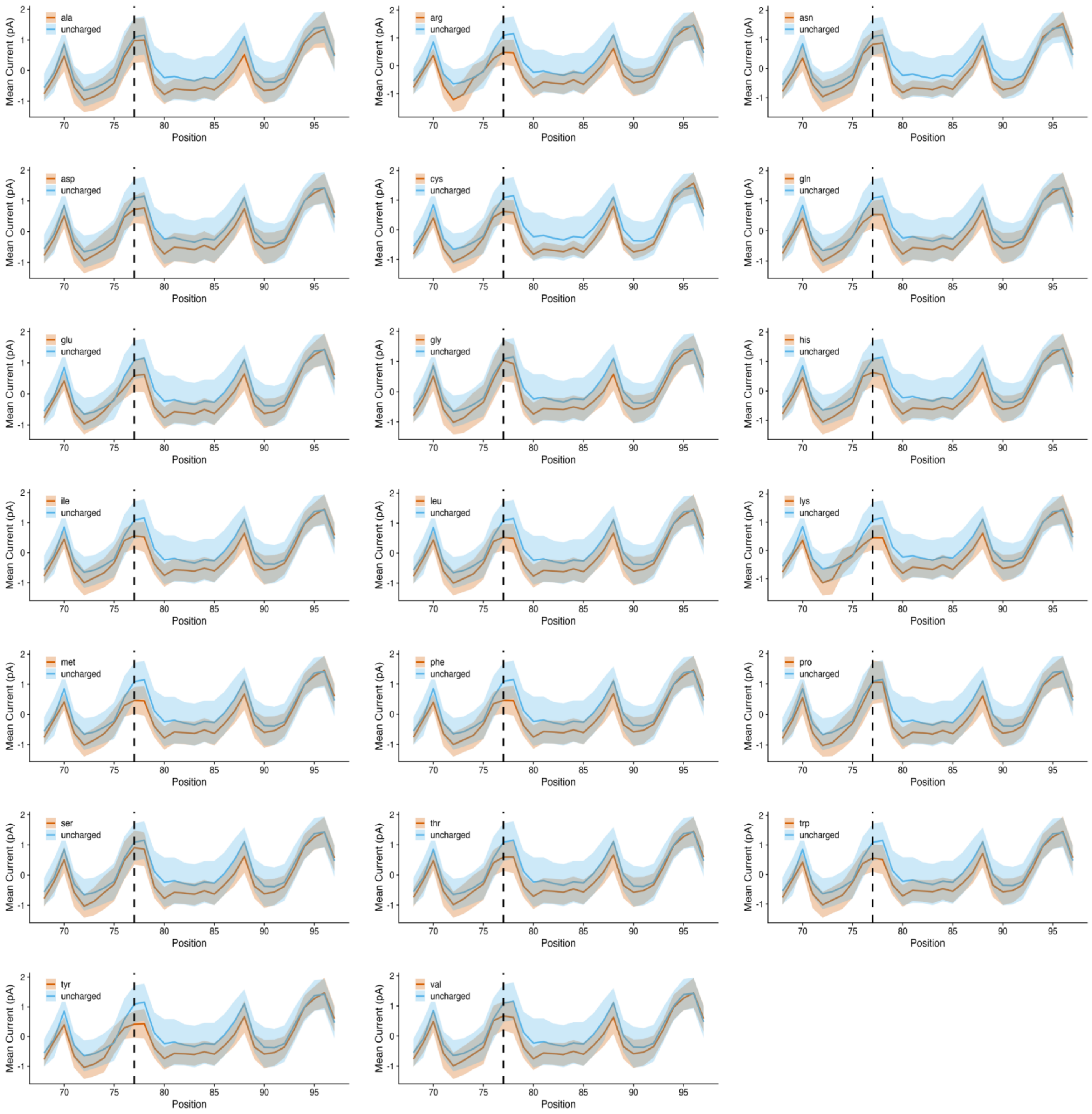

Supplementary Figure 9.

Normalized signal intensity (current in picoamps) for synthetic tRNA charged with 20 naturally-occurring amino acids using Flexizyme. Each panel plots the mean current in picoamps for an uncharged tRNA substrate in the central blue line, with the shaded blue area representing the standard deviation around the mean. Each aminoacylated comparison is plotted analogously in orange. The x-axis spans the same window of interest as in

Fig. 2, containing six nucleotides at the 3′ terminus of the tRNA, the CCA tail, the aminoacylated position (dashed line, included as an extra nucleotide inserted at position 77 in the reference) and the entirety of the 3′ adapter sequence from nt 78 onward.

Figure S10

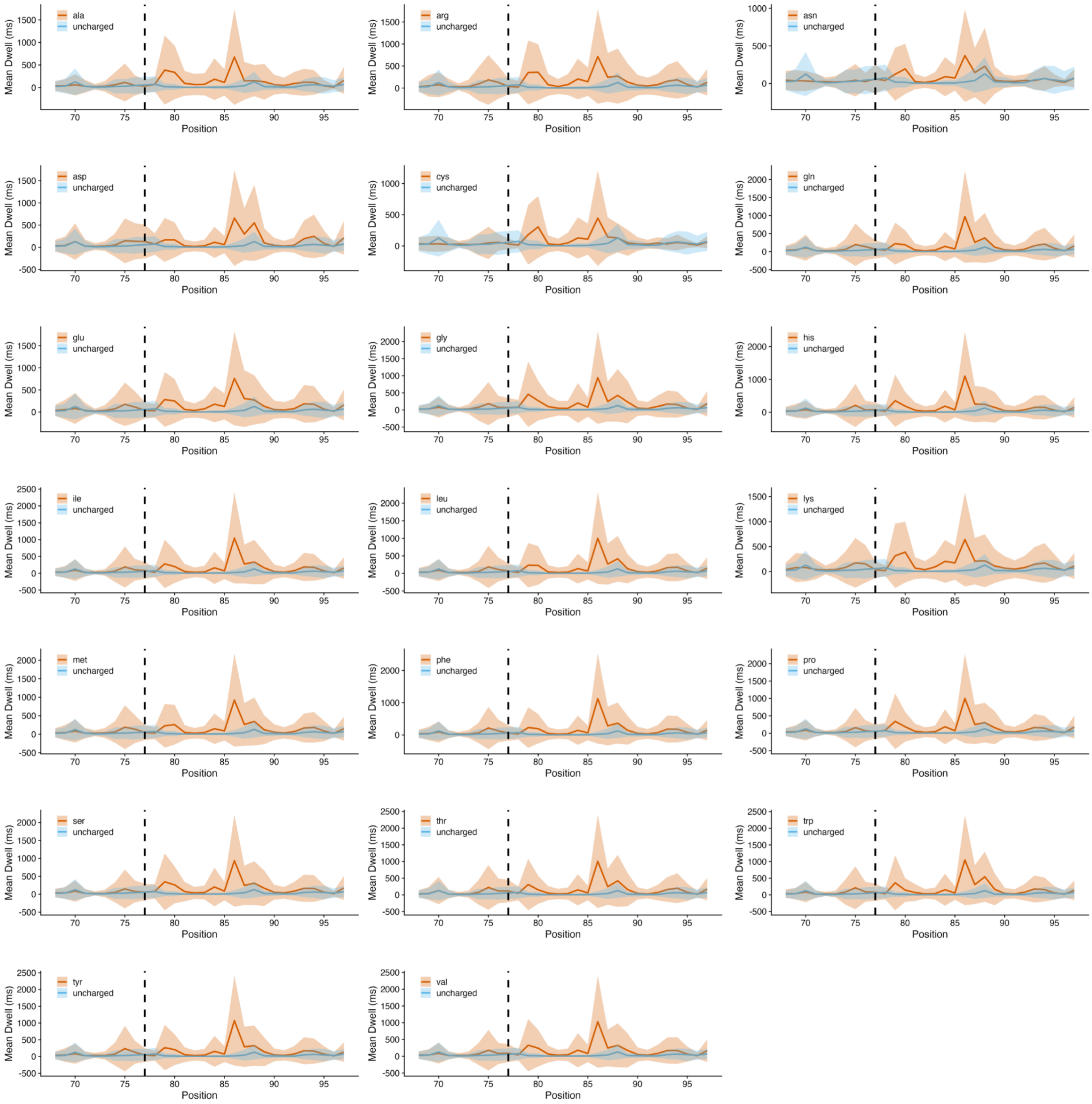

Supplementary Figure 10.

Mean dwell time for synthetic tRNA charged with 20 naturally-occurring amino acids using the Flexizyme. Each panel plots the mean dwell in milliseconds at each nucleotide in an uncharged tRNA substrate in the central blue line, with the shaded blue area representing the standard deviation around the mean. Each aminoacylated comparison is plotted analogously in orange. The x-axis spans the same window of interest as in **Fig. 2**,

containing six nucleotides at the 3′ terminus of the tRNA, the CCA tail, the aminoacylated position (dashed line, included as an extra nucleotide inserted at position 77 in the reference) and the entirety of the 3′ adapter sequence from nt 78 onward.
