# Supplementary material for: Nanopore sequencing of intact aminoacylated tRNAs
Source: Nat Commun. 2025 Aug 20;16:7781. doi: 10.1038/s41467-025-62545-9 (PMC12368100; doi:10.1038/s41467-025-62545-9)
Supplement: Supplementary file 2 — Reporting Summary [file 41467_2025_62545_MOESM2_ESM.pdf]

nature portfolio

Corresponding author(s): Laura White; Jay Hesselberth

Last updated by author(s): Jun 23, 2025

Reporting Summary

Nature Portfolio wishes to improve the reproducibility of the work that we publish. This form provides structure for consistency and transparency in reporting. For further information on Nature Portfolio policies, see our Editorial Policies and the Editorial Policy Checklist.

Statistics

For all statistical analyses, confirm that the following items are present in the figure legend, table legend, main text, or Methods section.

n/a	Confirmed	
☐	☒	The exact sample size (n) for each experimental group/condition, given as a discrete number and unit of measurement
☐	☒	A statement on whether measurements were taken from distinct samples or whether the same sample was measured repeatedly
☐	☒	The statistical test(s) used AND whether they are one- or two-sided<br>*Only common tests should be described solely by name; describe more complex techniques in the Methods section.*
☐	☒	A description of all covariates tested
☐	☒	A description of any assumptions or corrections, such as tests of normality and adjustment for multiple comparisons
☐	☒	A full description of the statistical parameters including central tendency (e.g. means) or other basic estimates (e.g. regression coefficient) AND variation (e.g. standard deviation) or associated estimates of uncertainty (e.g. confidence intervals)
☐	☒	For null hypothesis testing, the test statistic (e.g. F, t, r) with confidence intervals, effect sizes, degrees of freedom and P value noted<br>*Give P values as exact values whenever suitable.*
☒	☐	For Bayesian analysis, information on the choice of priors and Markov chain Monte Carlo settings
☒	☐	For hierarchical and complex designs, identification of the appropriate level for tests and full reporting of outcomes
☐	☒	Estimates of effect sizes (e.g. Cohen's d, Pearson's r), indicating how they were calculated

Our web collection on statistics for biologists contains articles on many of the points above.

Software and code

Policy information about availability of computer code

Data collection	Nanopore sequencing was performed using ONT MinKNOW software v24.06.10 on PromethION P2 Solo and P2i instruments. Basecalling was conducted using Dorado v0.7.2 with the “super high accuracy” rna004_130bps_sup v5.0.0 model and --emit-moves flag. Raw signal data were stored in POD5 format. Sequence alignment was performed using BWA-MEM v0.7.16 and samtools v1.21 was used to retain and process move table information for downstream analyses.
Data analysis	Signal features (e.g., dwell time, ionic current) were extracted using the Remora v3.2 API and analyzed in R and Python. Classification models were trained and validated using Remora, with aminoacylation state treated as a synthetic modification. Statistical comparisons (e.g., Z-tests with Benjamini–Hochberg correction) and F1 score calculations were performed in Python/R. A complete Snakemake pipeline and custom analysis scripts are available at https://github.com/rnabioco/aa-tRNA-seq.

For manuscripts utilizing custom algorithms or software that are central to the research but not yet described in published literature, software must be made available to editors and reviewers. We strongly encourage code deposition in a community repository (e.g. GitHub). See the Nature Portfolio guidelines for submitting code & software for further information.

Data

Policy information about availability of data

All manuscripts must include a data availability statement. This statement should provide the following information, where applicable:

- Accession codes, unique identifiers, or web links for publicly available datasets
- A description of any restrictions on data availability
- For clinical datasets or third party data, please ensure that the statement adheres to our policy

Raw nanopore sequencing data generated in this study has been deposited as POD5 files in the European Nucleotide Archive under accession number PRJEB90828. Per-read current and dwell time metrics for synthetic tRNA libraries are available on Zenodo under DOI 10.5281/zenodo.14194755. Source data supporting the findings of this study are provided with this paper.

Research involving human participants, their data, or biological material

Policy information about studies with human participants or human data. See also policy information about sex, gender (identity/presentation), and sexual orientation and race, ethnicity and racism.

Reporting on sex and gender	n/a
Reporting on race, ethnicity, or other socially relevant groupings	n/a
Population characteristics	n/a
Recruitment	n/a
Ethics oversight	n/a

Note that full information on the approval of the study protocol must also be provided in the manuscript.

Field-specific reporting

Please select the one below that is the best fit for your research. If you are not sure, read the appropriate sections before making your selection.

[x] Life sciences [ ] Behavioural & social sciences [ ] Ecological, evolutionary & environmental sciences

For a reference copy of the document with all sections, see nature.com/documents/nr-reporting-summary-flat.pdf

Life sciences study design

All studies must disclose on these points even when the disclosure is negative.

Sample size	No statistical methods were used to predetermine sample sizes. For synthetic tRNA ligation and sequencing experiments, sample sizes were chosen based on standard practices in RNA biochemistry and nanopore method development. For biological tRNA libraries, sample sizes were constrained by the throughput and availability of Oxford Nanopore direct RNA sequencing (dRNA-seq) over the course of the study. Although flow cell performance and sequencing yields improved during the project, total read depth and multiplexing were limiting for logistical reasons, including flow cell availability and run time. We prioritized library complexity and signal clarity over large-scale replication. For nutrient and temperature stress studies, three biological replicates were used to capture reproducibility of aminoacylation and abundance effects across conditions. Observed signal differences (e.g., shifts in Remora ML scores and read dwell times) were large relative to variability, and consistent across replicates. These sample sizes are consistent with other studies using nanopore sequencing or northern blotting to quantify tRNA charging and decay dynamics.
Data exclusions	No data were excluded.
Replication	All key findings were independently replicated with consistent results. Reproducibility was confirmed through the use of multiple biological replicates and orthogonal validation approaches, including parallel chemical-charging northern blotting and nanopore sequencing to assess tRNA aminoacylation. No findings were excluded due to lack of replication.
Randomization	This study used laboratory yeast strains grown under defined conditions. Allocation to experimental groups (e.g., nutrient or temperature stress) was based on culture conditions and is not subject to randomization. This approach is standard for microbial systems and covariates were controlled through parallel processing of matched cultures.
Blinding	Blinding was not relevant to this study. Experimental conditions were predefined and data collection involved quantitative molecular assays on yeast cultures processed in parallel.

Reporting for specific materials, systems and methods

We require information from authors about some types of materials, experimental systems and methods used in many studies. Here, indicate whether each material, system or method listed is relevant to your study. If you are not sure if a list item applies to your research, read the appropriate section before selecting a response.

Materials & experimental systems

n/a	Involved in the study
☐	☐ Antibodies
☐	☐ Eukaryotic cell lines
☐	☐ Palaeontology and archaeology
☐	☐ Animals and other organisms
☐	☐ Clinical data
☐	☐ Dual use research of concern
☐	☐ Plants

Methods

n/a	Involved in the study
☒	☐ ChIP-seq
☒	☐ Flow cytometry
☒	☐ MRI-based neuroimaging

Antibodies

Antibodies used	n/a
Validation	n/a

Eukaryotic cell lines

Policy information about cell lines and Sex and Gender in Research

Cell line source(s)	n/a; budding yeast
Authentication	deletions verified by PCR
Mycoplasma contamination	n/a
Commonly misidentified lines (See ICLAC register)	n/a

Palaeontology and Archaeology

Specimen provenance	n/a
Specimen deposition	n/a
Dating methods	n/a

☐ Tick this box to confirm that the raw and calibrated dates are available in the paper or in Supplementary Information.

Ethics oversight	n/a

Note that full information on the approval of the study protocol must also be provided in the manuscript.

Animals and other research organisms

Policy information about studies involving animals; ARRIVE guidelines recommended for reporting animal research, and Sex and Gender in Research

Laboratory animals	n/a
Wild animals	n/a
Reporting on sex	n/a
Field-collected samples	n/a
Ethics oversight	n/a

Note that full information on the approval of the study protocol must also be provided in the manuscript.

Clinical data

Policy information about clinical studies

All manuscripts should comply with the ICMJE guidelines for publication of clinical research and a completed CONSORT checklist must be included with all submissions.

Clinical trial registration	n/a
Study protocol	n/a
Data collection	n/a
Outcomes	n/a

Dual use research of concern

Policy information about dual use research of concern

Hazards

Could the accidental, deliberate or reckless misuse of agents or technologies generated in the work, or the application of information presented in the manuscript, pose a threat to:

No	Yes	
☒	☐	Public health
☒	☐	National security
☒	☐	Crops and/or livestock
☒	☐	Ecosystems
☒	☐	Any other significant area

Experiments of concern

Does the work involve any of these experiments of concern:

No	Yes	
☒	☐	Demonstrate how to render a vaccine ineffective
☒	☐	Confer resistance to therapeutically useful antibiotics or antiviral agents
☒	☐	Enhance the virulence of a pathogen or render a nonpathogen virulent
☒	☐	Increase transmissibility of a pathogen
☒	☐	Alter the host range of a pathogen
☒	☐	Enable evasion of diagnostic/detection modalities
☒	☐	Enable the weaponization of a biological agent or toxin
☒	☐	Any other potentially harmful combination of experiments and agents

Plants

Seed stocks	n/a
Novel plant genotypes	n/a
Authentication	n/a
